# In Vitro and In Vivo Toxicometabolomics of the Synthetic Cathinone PCYP Studied by Means of LC-HRMS/MS

**DOI:** 10.3390/metabo12121209

**Published:** 2022-12-02

**Authors:** Selina Hemmer, Lea Wagmann, Benedikt Pulver, Folker Westphal, Markus R. Meyer

**Affiliations:** 1 Department of Experimental and Clinical Toxicology, Institute of Experimental and Clinical Pharmacology and Toxicology, Center for Molecular Signaling (PZMS), Saarland University, 66421 Homburg, Germany; 2 State Bureau of Criminal Investigation Schleswig-Holstein, 24116 Kiel, Germany

**Keywords:** toxicometabolomics, PCYP, LC-HRMS, untargeted metabolomics

## Abstract

Synthetic cathinones are one important group amongst new psychoactive substances (NPS) and limited information is available regarding their toxicokinetics and -dynamics. Over the past few years, nontargeted toxicometabolomics has been increasingly used to study compound-related effects of NPS to identify important exogenous and endogenous biomarkers. In this study, the effects of the synthetic cathinone PCYP (2-cyclohexyl-1-phenyl-2-(1-pyrrolidinyl)-ethanone) on in vitro and in vivo metabolomes were investigated. Pooled human-liver microsomes and blood and urine of male Wistar rats were used to generate in vitro and in vivo data, respectively. Samples were analyzed by liquid chromatography and high-resolution mass spectrometry using an untargeted metabolomics workflow. Statistical evaluation was performed using univariate and multivariate statistics. In total, sixteen phase I and one phase II metabolite of PCYP could be identified as exogenous biomarkers. Five endogenous biomarkers (e.g., adenosine and metabolites of tryptophan metabolism) related to PCYP intake could be identified in rat samples. The present data on the exogenous biomarker of PCYP are crucial for setting up analytical screening procedures. The data on the endogenous biomarker are important for further studies to better understand the physiological changes associated with cathinone abuse but may also serve in the future as additional markers for an intake.

## 1. Introduction

In clinical and forensic toxicology, knowledge about the toxicometabolomics of drugs of abuse (DOAs) is important not only for reliable confirmation of a DOA intake by patients but also for their risk assessment in general [1]. Such knowledge is particularly important when the DOA itself can no longer be detected and metabolites or endogenous biomarkers are the only targets for their detection. At the end of 2020, the European Monitoring Centre for Drugs and Drug Addiction (EMCDDA) reported around 830 new psychoactive substances (NPS), including 156 synthetic cathinones [2]. Due to the structural diversity of NPS and the lack of toxicokinetic information (including metabolic fate), the detection of an intake by patients is an analytical challenge in clinical and forensic toxicology [3,4]. Furthermore, the fluctuating compounds of NPS available on the market make it difficult to regulate them and to evaluate sufficient risk assessment for each compound. 

Between 2019 and 2022, 29 synthetic cathinones have been identified for the first time [5]. They are classified as stimulants or amphetamine-type stimulants [6,7]. The pharmacological effects of the different derivates depend on the type of substituents and their location. In preclinical studies, two ways on interaction with monoamine transporters were demonstrated: monoamine transporter blockers such as cocaine or monoamine transporter substrates stimulated the release of neurotransmitters such as amphetamine and MDMA [8,9]. 

The synthetic cathinone PCYP (2-cyclohexyl-1-phenyl-2-pyrrolidin-1-yl-ethan-1-one) was first detected in March 2019 in the U.S., and in Europe the first case report was published 2020 [5]. Due to the presence of the lipophilic and bulky cyclohexyl ring, PCYP exhibited an up to twofold stronger interaction with dopamine transporters in vitro compared to alpha-pyrrolidinovalerophenone (α-PVP). Therefore, it shows stronger dopaminergic stimulation and higher addictive potential [10]. This biochemical reaction led to desired effects such as stimulation and euphoria, but also to adverse effects including restlessness, anxiety, psychosis, tachycardia, and hyperthermia [5]. So far, no data are available about the metabolic fate of PCYP and the impact of PCYP on endogenous metabolic pathways. To date, only one case report of PCYP intake in Europe has been published [5]. It cannot be excluded that there is, was, and will be more extensive distribution. To uncover such abuse, screening procedures need to be up to date, which is often not possible in cases where the urinary screening targets are not known.

In recent years, toxicometabolomics, a subdiscipline of metabolomics, has increasingly gained interest in the study of the toxicokinetic and -dynamic DOAs [3,11,12,13,14,15,16]. The application of untargeted toxicometabolomics may allow researchers to find exogenous biomarkers, such as new drug metabolites, and endogenous biomarkers. Not only could these be indicators of acute drug ingestion or sample manipulation, but they could also offer information on the mode of action of the drugs and consumption patterns or could be used to assess the severity of intoxication [17,18,19]. Due to the lack of authentic human samples, toxicometabolomic studies are often conducted using different in vitro and in vivo models, such as pooled human-liver microsomes (pHLMs), HepaRG cell lines, and/or rats [13,14,15]. 

Since data about neither the metabolic pathway of PCYP nor the impact on the metabolome are available, this study aimed to provide the metabolic profile in an in vitro model using pHLM incubation. In conducting an in vivo experiment providing rat plasma and rat urine, the endogenous response to an acute PCYP exposure should be revealed. Analysis will be conducted by liquid chromatography coupled with high-resolution tandem mass spectrometry (LC-HRM/MS) using an untargeted metabolomics workflow. The resulting data should enable us to overcome the analytical challenge in clinical and forensic toxicology to confirm patient intakes of PCYP and to understand its acute and chronic effects. 

## 2. Materials and Methods

### 2.1. Materials and Chemicals

PCYP hydrochloride was provided by the State Bureau of Criminal Investigation Schleswig-Holstein (E.U. project ADEBAR plus, Kiel, Germany) for research purposes. The chemical purity of >93% and the identity of the compound was verified by MS and nuclear magnetic resonance analysis. Ammonium formate, ammonium acetate, creatinine-d_3_, dipotassium phosphate, formic acid, D-glucose-1,2,3,4,5,6,6-d_7_, isocitrate dehydrogenase, isocitrate, magnesium chloride, palmitic acid-d_31_, superoxide dismutase, and tripotassium phosphate were obtained from Merck (Darmstadt, Germany). Acetonitrile, ethanol, methanol (all LC-MS grade), and NADP-Na_2_ were from VWR (Darmstadt, Germany). L-Tryptophan-d_5_ was obtained from Alsachim (Illkirch-Graffenstaden, France). 1-Palmitoyl-d_9_-2-palmitoyl-sn-glycero-3-PC and prostaglandin-E_3_-d_9_ were from Cayman Chemical (Ann Arbor, MI, USA). Water was purified with a millipore filtration unit (18.2 W × cm water resistance). pHLMs (20 mg microsomal protein × mL^−1^, 360 pmol total CYP/mg, 26 donors) were obtained from Corning (Amsterdam, The Netherlands). After delivery, pHLMs were thawed at 37 °C, aliquoted, snap-frozen in liquid nitrogen, and stored at −80 °C until use. 

### 2.2. Sample Preparation and Analysis of pHLM Incubation

According to published procedures [3,20], incubations using pHLMs were prepared as follows. PCYP was dissolved freshly in methanol and subsequently diluted with 0.1 M phosphate buffer to obtain the required concentrations. Incubations were performed using a final PCYP concentration of 0 (blank group) or 50 µM (PCYP group) and 1 mg protein mL^−1^ pHLM at 37 °C. The final incubation mixtures also contained 90 mM phosphate buffer, 5 mM isocitrate, 5 mM Mg^2+^, 1.2 mM NADP^+^, 200 U/mL superoxide dismutase, and 0.5 U mL^−1^ isocitrate dehydrogenase. A final incubation volume of 50 µL was obtained. The reaction was stopped after 60 min by adding 50 µL of ice-cold acetonitrile and then centrifuged for 2 min at 18,407× *g*. For each group, 5 replicates were prepared. Pooled-quality samples (QC group) were prepared by transferring 20 µL of each replicate incubation into one MS vial. QC samples were used for optimization of the peak-picking parameters and identification of significant features, as described below.

### 2.3. Study Design In Vivo

Ten adolescent male Wistar rats (Charles River, Sulzfeld, Germany) were housed in a controlled environment (temperature 22 °C, humidity 57 ± 2%, and 12 h light/dark cycle). Studies were approved by an ethics committee (33/2019-Landesamt für Verbraucherschutz, Saarbrücken, Germany). A single dose of 2 mg/kg body weight (BW) PCYP was administered to five rats as aqueous suspension by gastric intubation. Five control rats were administrated only with water. During the study, rats were housed in metabolism cages for 24 h, having water ad libitum. Animal general health aspects were assessed at the time points 30, 60, 120, 360 min, and 24 h after intake. 

### 2.4. Sample Collection In Vivo

Blood samples of 0.5 mL were collected from each rat one hour after administration. For blood sampling, animals were anesthetized with diethyl ether and blood was taken from the *Vena caudalis mediana* using a heparin-coated syringe. Blood samples were centrifuged (1503× *g*, 5 min, 24 °C) and plasma was removed and immediately stored at −80 °C until analysis. Urine was collected separately from the feces over a period of 24 h after administration, aliquoted, frozen, and stored at −80 °C until use.

### 2.5. Sample Preparation and Analysis of Rat Blood Plasma and Rat Urine

According to Manier and Meyer [21], blood plasma samples were prepared as follows: an amount of 50 µL plasma was transferred into a reaction tube and precipitated using 200 µL of a mixture of methanol and ethanol (1:1, *v*/*v*). The mixture contained 48 µM L-tryptophan-d_5_, 8.6 µM creatinine-d_3_, 34.8 µM palmitic acid-d_31_, and 53.4 µM D-glucose-d_7_ as internal standard. Samples were shaken for 2 min at 2000 rpm and subsequently centrifuged at 21,130× *g* and 2 °C for 30 min. A volume of 150 µL of the supernatant was transferred into a new reaction tube and evaporated to dryness using a vacuum centrifuge at 1400 rpm and 24 °C for 20 min. The obtained residues were reconstituted in 50 µL of a mixture of acetonitrile and methanol (70:30, *v*/*v*).

Based on Hemmer et al. [15], urine samples were centrifuged at 13,523× *g* at 4 °C for 10 min. Volumes of 100 µL of urine were transferred into reaction tubes and 400 µL methanol, including 48 µM L-tryptophan-d_5_, 8.6 µM creatinine-d_3_, 34.8 µM palmitic acid-d_31_, and 53.4 µM D-glucose-d_7_ as internal standard, was added. Samples were cooled to −20 °C for 20 min and then centrifuged at 13,523× *g* and 4 °C for 10 min. An amount of 350 µL of the supernatant was transferred into a new reaction tube and evaporated to dryness using a vacuum centrifuge at 1400 rpm and 24 °C. The obtained residues were reconstituted in 50 µL of a mixture of acetonitrile and methanol (70:30, *v*/*v*).

Pooled QC samples were prepared by transferring 50 µL of each sample into one MS vial. These QC samples were also used for optimization of the peak-picking parameters and identification of significant features, as described below (QC group). QC samples, and each sample of control rats (water administration) and PCYP rats (PCYP administration) were stored until use at −80 °C.

### 2.6. LC-HRMS Apparatus

According to published procedures [3,15,20], analyses were performed using a Thermo Fisher Scientific (TF, Dreieich, Germany) Dionex UltiMate 3000 RS pump consisting of a degasser, a quaternary pump, and an UlitMate Autosampler, coupled with a TF Q Exactive Plus equipped with a heated electrospray ionization (HESI)-II source. Performance of the columns and the mass spectrometer was tested using a test mixture described by Maurer et al. [1,22]. Gradient reversed-phase (RP) elution was performed on a TF Accucore Phenyl-Hexyl column (100 mm × 2.1 mm, 2.6 µm) and hydrophilic interaction chromatography (HILIC) elution using a Merck (Darmstadt, Germany) SeQuant ZIC HILIC (150 mm × 2.1 mm, 3.5 µm). The mobile phase for the RP chromatography consisted of 2 mM aqueous ammonium formate containing acetonitrile (1%, *v*/*v*) and formic acid (0.1%, *v*/*v*, pH 3, eluent A), as well as 2 mM ammonium formate solution with acetonitrile:methanol (1:1, *v*/*v*) containing water (1%, *v*/*v*) and formic acid (0.1%, *v*/*v*, eluent B). The flow rate was set from 0 to 10 min to 500 µL/min and from 10 to 13.5 min to 800 µL/min using the following gradient: 0–1 min hold 99% A, 1–10 min to 1% A, 10–11.5 min hold 1% A, and 11.5–13.5 min hold 99% A. The gradient elution for HILIC was performed using aqueous ammonium acetate (200 mM, eluent C) and acetonitrile containing formic acid (0.1%, *v*/*v*, eluent D). The flow rate was set to 500 µL/min using the following gradient: 0–1 min hold 2% C, 1–5 min to 20% C, 5–8.5 min to 60% C, 8.5–10 min hold 60% C, and 10–12 min hold 2% C. Injection volume was set to 1 µL for all samples. For preparation and cleaning of the injection system, isopropanol:water (90:10, *v*/*v*) was used. The following settings were used: wash volume, 100 µL; wash speed, 4000 nL/s; loop wash factor, 2. Column temperature for every analysis was set to 40 °C, maintained by a Dionex UltiMate 3000 RS analytical column heater. HESI-II source conditions were as follows: ionization mode, positive or negative; sheath gas, 60 AU; auxiliary gas, 10 AU; sweep gas, 3 AU; spray voltage, 3.5 kV in positive and −4.0 kV in negative mode; heater temperature 320 °C; ion transfer capillary temperature, 320 °C; and S-lens RF level, 50.0. Mass spectrometry for untargeted metabolomics was performed according to a previously optimized workflow [3,23]. The settings for full-scan (FS) data acquisition were as follows: resolution 140,000 at *m*/*z* 200; microscan, 1; automatic gain control (AGC) target, 5e5; maximum injection time, 200 ms; scan range, *m*/*z* 50–750; spectrum data type; centroid. All study samples were analyzed in randomized order to avoid potential analyte instability or instrument performance potentially confounding data interpretation. Additionally, one QC injection was performed every five samples to monitor batch effects, as described by Wehrens et al. [24]. Significant features were subsequently identified using PRM. Settings for PRM data acquisition were as follows: resolution, 35,000 at *m*/*z* 200; microscans, 1; AGC target, 5e5; maximum injection time, 200 ms; isolation window, *m*/*z* 1.0; collisions energy (CE), 10, 20, 35, or 40 eV; spectrum data type, centroid. The inclusion list contained the monoisotopic masses of all significant features and a time window of their retention time ± 60 s. TF Xcalibur software version 3.0.63 was used for data handling.

### 2.7. Data Processing and Statistical Analysis

Data processing for untargeted metabolomics was performed in an R environment according to previously published workflows [15,23]. TF LC-HRMS/MS RAW files were converted into mzXML files using ProteoWizard [25]. XCMS parameters were optimized using a previously developed strategy, as mentioned by Manier et al. [23]. Peak-picking and alignment parameters are summarized in Table S1. Peak picking was performed using XCMS in an R environment [26,27], and the R package CAMERA [28] was used for the annotation of adducts, artifacts, and isotopes. Feature abundances with a value of zero were replaced by the lowest-measured abundance as a surrogate limit of detection and the whole dataset was then log 10 transformed [24]. Normalization was performed for urine samples using the area of endogenous creatinine from those samples analyzed using HILIC column and positive ionization mode. For plasma samples, normalization was performed using the area of L-tryptophane-d_5_. Significant changes in features between control and PCYP respectively blank and PCYP groups were assumed after Welch’s two-sample *t*-test and Bonferroni correction for pHLM [29]; *p*-value < 0.01 for urine, and *p*-value < 0.05 for plasma. Principal component analysis (PCA) and hierarchal clustering were used to investigate patterns in the datasets. For pHLM, t-distributed stochastic neighborhood embedding (t-SNE) [30,31] was used instead of PCA. Names for features were adopted from XCMS using “M” followed by rounded mass and “T” followed by the retention time in seconds. After visual inspection of the extracted ion chromatograms (EIC) of significant features, based on the peak shape quality, the significant features were divided into true and false features [20]. The R scripts can be found on GitHub (https://github.com/sehem/PCYP_Metabolomics.git) and the mzXML files used in this study are available via Metabolights (study identifier MTBLS6469).

### 2.8. Identification of Significant Features

Significant features were identified by recording MS/MS spectra using the PRM method mentioned above. After conversion to mzXML format using ProteoWizard [25], spectra were imported to NIST MS Search version 2.3 Library. The settings for library and MS/MS search were used according to published procedures [14,15,20]. Metabolites of the synthetic cathinone PCYP were tentatively identified by interpreting their spectra in comparison to that of the parent compound. Identified features were classified on the different levels of identification according to the metabolomics standards initiative (MSI) [32].

## 3. Results and Discussion

### 3.1. Study Design

Two different models were used to investigate the toxicometabolomics of the synthetic cathinone PCYP via an untargeted approach. The in vitro model used is common in drug metabolism studies due to its ease of use and low variability [33]. Rat, as in vivo model, was used to investigate the impact of the synthetic cathinone on the rat metabolome. In comparison to cell lines, plasma or urine samples are very complex since the metabolome can also be affected by, for example, food, the microbiome, and drugs used to anesthetize animals [34]. Due to the complexity and influence of the metabolome, animal models are well-suited for studying changes in the metabolome compared to human studies. Animal studies can be performed under standardized and comparable conditions. For example, animals are subject to a uniform sleep–wake rhythm, and they can be kept under the same conditions and obtain the same water and food. Due to their very low genetic variability, it is also possible to obtain reliable results with significantly fewer animals compared to human clinical studies. Compared to in vitro studies, which often only represent certain cell components, cells, or organs, in vivo studies offer the possibility to provide an insight into the whole organism. Besides elucidation of the endogenous response, urine also offers the possibility to analyze for drug metabolites. The knowledge about xenobiotic metabolic pathways is essential for clinical and forensic toxicology to develop suitable analytical screening procedures to detect consumption [5,8,9]. Compared to conventional methods for analyzing metabolic pathways, an untargeted urinary toxicometabolomics approach allows for the detection of metabolites which might be overlooked as they are not expected [3,14,35]. Besides toxicokinetics, there is limited information available about the mode of action of synthetic cathinones, especially of PCYP. This is where the blood plasma comes into play. Plasma samples are of interest with respect to changes in endogenous metabolites that may be affected by the intake of drugs of abuse. 

### 3.2. Untargeted Data Processing and Statistical Analysis 

Univariate statistics were performed using volcano plot. False-positive results were prevented by using Bonferroni correction [29] for pHLM-derived data, with *p*-value > 0.01 for urine-derived data, and *p*-value > 0.05 for plasma-derived data. Results of the identification of significant features and their level of identification in accordance with the MSI [32] are summarized in Tables S2–S4. Annotated isotopes by CAMERA were not further analyzed. Features were analyzed as described above using the PRM method, and MS^2^ spectra for PCYP metabolites are shown in Figure S8. For several features, no MS^2^ spectra could be recorded due to their low abundance.

Using the four different analytical methods (RP positive, RP negative, HILIC positive, HILIC negative), thirty features, containing eleven isotopes and one adduct, were found in total to be significant in pHLM incubation. Analysis using RP and HILIC and negative ionization mode did not reveal any significant changes. Rat plasma samples, which were taken 1 h after administration, revealed 17 metabolites and 3 isotopes using above-mentioned analytical methods. In urine samples, 122 significant features were found in total containing 16 isotopes and 1 adduct.

Besides univariate statistics, the different datasets were also evaluated regarding the results of multivariate statistics to identify the largest changing features and specific signatures in the data. Since multivariate statistics could only be performed if there were at least two significant features, no data were available for datasets containing no or only one significant feature. For all analyses and matrices, it can be shown that the PCYP and blank or control groups were distinct from each other (Figures S1–S3). Complementary to the scores plot, the loadings plot provided information about which metabolites had the greatest contribution to the separations between groups [36]. Thereby, it can be seen that especially PCYP itself and its metabolites lead to the separation of the individual groups. For data derived from the pHLM incubations (Figure S1), the variance in the first principal component was between 99 and 97% using RP and HILIC in positive ionization mode. These results indicated that the pHLM datasets were highly linear, revealing that the PCA is not suitable for those experiments where only the parent compound and its metabolites are detectable. Therefore, the patterns in the pHLM dataset were evaluated using t-SNE, which is a dimension reduction algorithm that visualizes similarities in datasets [31]. Results of the t-SNEs (Figure S4) showed similar cluster patterns for all analyses. This can be explained by the fact that data derived from pHLM incubations show low variability and only PCYP itself and its metabolites led to the separation of the two groups.

In addition to PCA, hierarchical clustering was also performed. In untargeted metabolomics studies, heat maps of hierarchical clustering can be used to discover clustering patterns in the datasets. For all analyses and matrices, the hierarchical clustering mostly revealed a high distance of samples from blank or control group to those from PCYP and QC groups (Figures S5–S7). However, there was an exception for urine samples separated by HILIC in positive ionization mode (Figure S7C). In this case, two QC samples were clearly separated from other data. Taking a closer look at these two runs, it was observed that the total ion chromatogram of these two samples showed a higher intensity than the other QCs, even though it was the same sample. Reasons for this remain unclear.

### 3.3. Metabolic Pathways of PCYP

**Figure 1:**
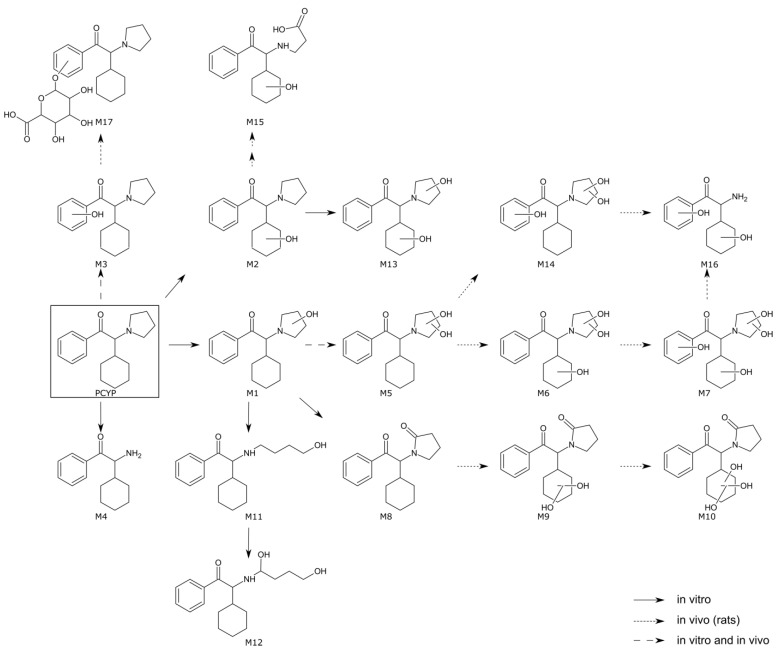
In vitro and in vivo metabolic pathways of PCYP. The parent compound is indicated by a black square, undefined hydroxylation positions are indicated by unspecific bonds. Metabolite identification numbers (M) match with the metabolites listed in Table S5.

The proposed metabolic pathways of PCYP in the in vitro and in vivo models are summarized in Figure 1. The MS^2^ spectra of all PCYP metabolites are shown in Figure S8. Table S5 provides a list of all metabolites in terms of their abundance in each column and matrix. Additionally, Table S5 includes the metabolite identification number (M), the calculated exact mass of the protonated molecule, and the elemental composition of all detected metabolites, respectively. The corresponding retention times of each metabolite for each column are given in Tables S2–S4 in the Supporting Information. Figures S9 and S10 show the reconstructed chromatograms of the most abundant metabolites in pHLM and rat urine.

In total, sixteen phase I and one phase II metabolite were found in all three matrices using the four different above-mentioned analytical methods. Not metabolized PCYP could only be detected in vitro but not in the in vivo samples. However, this was not surprising since the average elimination half-time of the structure analog α-PVP was reported to be <2.1 h in Sprague–Dawley rats after injection [37]. Regarding the in vitro phase I metabolism, PCYP was reduced by a *N*,*N*-bis-dealkylation (M4), which was also reported for α-PVP [38,39,40,41]. In accordance with previous publications, the pyrrolidine ring underwent biotransformation resulting in a mono- (M1) and dihydroxylation (M5), an oxo- (M8) as well as a ring-opened mono- (M11) and dihydroxy metabolite (M12) [3,42]. The opening of the pyrrolidine ring has also previously been observed for the two synthetic cathinones α-PBP and α-PEP and is most likely the result of hydroxylation at the ortho-position of the pyrrolidine ring, followed by a retrohemiaminal reaction [3]. The combination of hydroxylation on hexyl and pyrrolidine ring leading to a dihydroxylation (M13) was also detected in pHLM incubations. 

Nine phase I metabolites could be identified in vivo, amongst them the monohydroxylation at the benzyl-ring (M3) and the dihydroxylation at the pyrrolidine ring (M5). Additionally, a combination of di- (M9) and trihydroxylation (M10) on the hexyl ring and oxidation at the pyrrolidine ring was found in urine samples. The combination of hydroxylation on hexyl and pyrrolidine ring leading in a dihydroxylation (M13) was also detected. Tri- and tetrahydroxylation metabolites were found, resulting in a dihydroxylation on the pyrrolidine ring followed by a monohydroxylation on the hexyl (M6) and/or benzyl ring (M7, M14). Tetrahydroxylation led to a bis-*N*-dealkylation (M16). Another metabolite, which was only detected in urine samples, consisted of hydroxylation on the hexyl ring and pyrrolidine cleavage followed by oxidation to carboxylic acid (M15). The metabolites M5, M6, and M9 could also be observed in rat plasma. Regarding phase II metabolism, only the conjugation with glucuronic acid after hydroxylation of the benzyl ring (M17) could be observed. No other conjugates, such as glucuronic acid or sulfate, could be found. The lower abundance of phase II metabolites can be explained by the fact that drug-metabolizing enzymes such as cytochrome P450 or glucuronosyltransferases have different expressions and functions in different species. Therefore, significantly more phase I metabolites are formed in rat liver compared to humans, whereas more phase II metabolites are formed in humans [43,44,45,46].

Since the parent compound could no longer be detected in 24 h urine, analytical procedure should include these metabolites in addition to the parent compound, considering its probability of being not detectable in urine after lower doses or after sampling times later than 24 h after intake. Therefore, reference spectra need to be added to common MS databases to allow detection [22,47]. Nevertheless, authentic human samples are required to fill the gap between in vitro and in vivo assays and to reliably determine which metabolites are useful for screening procedures in humans. 

### 3.4. Effect of PCYP on the Rat Metabolome 

Since there is limited information available on the effects of NPS on the metabolome [11,48], untargeted toxicometabolomics have been increasingly used to study their toxicity-related pathways. Toxicometabolomics combines the detection and identification of endogenous and exogenous biomarker. This allows the determination of metabolites of the investigated substance in order to detect an intake by patients, as well as the identification of biomarkers that provide information on the effect of substances on the metabolome in only one experiment [49,50]. 

The complexity of the metabolome becomes visible by comparing the PCAs of the three investigated matrices (pHLM incubations, plasma samples, and urine samples) in this study (Figures S1–S3). Since the PCAs in pHLM are highly linear and only PCYP and its metabolites were identified as significant features, rat urine and rat plasma samples showed higher variability. In rat plasma samples collected 1 h after administration, three significantly altered metabolites could be identified by MSI level 2 or 3 [32]. In PCYP-treated rats, adenosine was significantly increased. Adenosine is a ubiquitous nucleoside and is consequently involved in many biological processes as a component of DNA or RNA. For example, it plays an important role in energy transfer as adenosine diphosphate (ADP) or -triphosphate (ATP). As cyclic adenosine monophosphate (cAMP), it also plays a role in signal transduction. Furthermore, adenosine itself is both a neurotransmitter and a potent vasodilator [51]. Altered adenosine levels after acute or chronic consumption of drugs of abuse and psychostimulants have already been reported in several studies [52,53,54,55,56]. Other studies have shown that high levels of adenosine induce sleep in rats [57,58,59]. During the monitoring of the animal general health aspects at the time point 30 min, 60 min, 120 min, 360 min, and 24 h, no significant change in the sleep behavior could be observed between the two groups. Another endogenous metabolite that was significantly increased in the plasma of PCYP-treated rats was 3-methyladipic acid. 3-methyladipic acid itself is a metabolite of the catabolism of the naturally occurring phytanic acid and is involved in biological processes such as lipid peroxidation, fatty acid metabolism, cell signaling, and the lipid metabolism pathway [51]. Quinoline-2-ol was also significantly increased in rat plasma as well as in rat urine of PCYP-treated rats. However, the biological significance of this metabolite is currently unclear.

Urine is distinguished from plasma by being easily collected, rich in metabolites, and capable of reflecting imbalances in all biochemical pathways within an organism [60]. It is well-suited for the identification of novel exogenous drug metabolites or endogenous biomarkers indicative for drug ingestion unless they are not exclusively excreted in feces. In this study, ten PCYP metabolites could be identified in rat urine collected 24 h after administration, which are described in detail above. In addition to quinoline-2-ol, which was also significantly present in rat urine, three other metabolites were identified in rat urine that did not belong to PCYP. Daidzein, is an isoflavone and is known as a biomarker for the consumption of soybeans and other soy products [51]. It was significantly increased in urine of PCYP-treated rats. Since the rats had only water and no food available in their metabolic cage after substance administration, this finding cannot be associated with the consumption of PCYP. The last two metabolites which were significantly changed in rat urine belong to the tryptophan metabolism. Kynurenic acid was significantly decreased in PCYP-treated rats. In the tryptophan metabolism, kynurenic acid is a metabolite of L-kynurenine and also known as neuroprotective agent. Several studies reported a reduced kynurenic acid in mood disorders such as depressive or bipolar disorders [61,62,63]. Dihydroxyquinoline was increased in PCYP-treated rats. In tryptophan metabolism, 4,6-dihydroxyquinoline and 4,8-dihydroxyquinoline are degradation products of hydroxykynurenamine (HMDB). This observation suggests that PCYP induces the tryptophan metabolism. Kolanos et al. demonstrated in an in vitro experiment, that PCYP, due to its structure, shows strong dopaminergic stimulation [10]. Based on these two observations, it can be hypothesized that synthetic cathinones such as PCYP may directly affect neurotransmission, and thereby affect important metabolic pathways such as tryptophan metabolism. Since the present study provides only a snapshot of the metabolome in rats and only two metabolites of the tryptophan metabolism could be identified, further studies are required to obtain a reliable conclusion. 

Furthermore, it is important to keep in mind that a direct correlation to humans is not possible. The few altered endogenous metabolites in this study could only be partly explained regarding their general function in mammals. Since it is very difficult to make a reliable conclusion about a specific pathway based on one or two metabolites, further studies are needed. These studies should be based on a targeted metabolomics approach on the alteration of the tryptophan metabolism after PCYP intake.

## 4. Conclusions

The present study provides a snapshot on the altered metabolic pathway after acute intake of the synthetic cathinone PCYP. Using untargeted toxicometabolomics, sixteen phase I and one phase II metabolites of PCYP could be identified in vitro and in vivo. The main metabolic reaction in rat urine was the dihydroxylation on the pyrrolidine ring followed by mono- and/or dihydroxylation on the benzyl and/or hexyl ring. Regarding phase II metabolism, only the glucuronidation after hydroxylation on the benzyl ring could be observed. Since there are no data available regarding the metabolic pathways of PCYP, the identified metabolites in this study could be used for detection of PCYP intake. 

Additionally, five endogenous metabolites could be identified as being significantly altered after PCYP intake. Particular attention should be paid to the two metabolites which are involved in tryptophan metabolism. Since there are many more metabolites involved in this metabolism, further studies are required to confirm this observation. The results of this study demonstrate how the use of toxicometabolomic workflows can overcome conventional screening methods to identify metabolites and endogenous biomarkers that would not be expected. Thus, the knowledge obtained from this study of the rat metabolome can be applied to similar compounds and provide insights into the effects of the compound (class) on an organism. Overall, this study contributes to the understanding of the influence of synthetic cathinones, especially PCYP, on the mammalian metabolome. However, further studies are essential to support the results of this study and to investigate the applicability to humans. 

## Data Availability

The R scripts can be found on GitHub (https://github.com/sehem/PCYP_Metabolomics.git) and the mzXML files used in this study are available via Metabolights (www.ebi.ac.uk/metabolights/MTBLS6469 (accessed on 17 November 2022)).

## Supplementary Materials

The following supporting information can be downloaded at: https://www.mdpi.com/article/10.3390/metabo12121209/s1, Table S1: Overview of the peak-picking and alignment parameters used for preprocessing for the reversed-phase (RP) and hydrophilic interaction chromatography (HILIC) column and the respective matrices. Pos = positive, neg = negative, ppm = allowed ppm deviation of mass traces for peak picking, snthresh = signal to noise threshold, mzdiff = minimum difference in *m*/*z* for two peaks to be considered as separate, prefilter 1 = minimum of scan points, prefilter 2 = minimum abundance, bw = bandwidth for grouping of peaks across separate chromatograms; Table S2: Overview of the significant features using reversed-phase (RP) and hydrophilic interaction chromatography (HILIC) column in pooled human-liver microsome (pHLM) incubation. Features are sorted according to *m*/*z* values, followed by the polarity, the retention time (RT) for the corresponding column in seconds (sec), identity, and the identification level according to MSI. Hyphen (-) means that the feature was not significant using the corresponding column; Table S3: Overview of the significant features using reversed-phase (RP) and hydrophilic interaction chromatography (HILIC) column in rat. Features are sorted according to *m*/*z* values, followed by the polarity, the retention time (RT) for the corresponding column in seconds (sec), identity, and the identification level according to MSI. Hyphen (-) means that the feature was not significant using the corresponding column; Table S4: Overview of the significant features using reversed-phase (RP) and hydrophilic interaction chromatography (HILIC) column in rat urine. Features are sorted according to *m*/*z* values, followed by the polarity, the retention time (RT) for the corresponding column in seconds (sec), identity, and the identification level according to MSI. Hyphen (-) means that the feature was not significant using the corresponding column; Table S5: Detected PCYP metabolites using reversed-phase (RP) and hydrophilic interaction chromatography (HILIC) column in their corresponding matrices namely pooled human-liver microsomes (H), rat urine (U), and rat plasma (P) in which the metabolites could be detected. Metabolite identification numbers (ID) match with the labeling of the structure in Figure 1. For each metabolite, the calculated exact mass of the protonated molecule and elemental composition are given. Hyphen (-) means that the metabolite was not significant in any matrix of the respective column; Figure S1: Results of scores of principal component analysis of pooled human-liver microsome samples after analysis using reversed-phase (RP) and hydrophilic interaction chromatography (HILIC) in positive ionization mode. A = RP pos, B = HILIC pos; Figure S2: Results of scores of principal component analysis of rat urine samples after analysis using reversed-phase (RP) and hydrophilic interaction chromatography (HILIC) in positive and negative ionization mode. A = RP pos, B = RP neg, C = HILIC pos, D = HILIC neg; Figure S3: Results of scores of principal component analysis of rat plasma samples after analysis using reversed-phase (RP) and hydrophilic interaction chromatography (HILIC) in positive and negative ionization mode. A = PH pos, B = HILIC pos, C = HILIC neg; Figure S4: Results of t-distributed stochastic neighborhood embedding (t-SNE) of pooled human-liver microsome samples after analysis using reversed-phase (RP) and hydrophilic interaction chromatography (HILIC) in positive ionization mode. A = RP pos, B = HILIC pos; Figure S5: Results of heat map of hierarchical clustering of pooled human-liver microsome samples after analysis using reversed-phase (RP) and hydrophilic interaction chromatography (HILIC) in positive ionization mode. A = RP pos, B = HILIC pos; Figure S6: Results of heat map of hierarchical clustering of rat urine samples after analysis using reversed-phase (RP) and hydrophilic interaction chromatography (HILIC) in positive and negative ionization mode. A = RP pos, B = RP neg, C = HILIC pos, D = HILIC neg; Figure S7: Results of heat map of hierarchical clustering of rat plasma samples after analysis using reversed-phase (RP) and hydrophilic interaction chromatography (HILIC) in positive and negative ionization mode. A = RP pos, B = HILIC pos, C = HILIC neg; Figure S8: LC-HRMS/MS spectra of the PCYP metabolites detected in positive ionization mode. Fragments with accurate mass, calculated elemental formula, and mass error value in parts per million (ppm); Figure S9: Reconstructed ion chromatogram of *m*/*z* 288.1958 after analysis of one QC sample of pooled human-liver microsome in full scan in positive ionization mode using hydrophilic interaction chromatography (HILIC). Metabolite identification number (M) match with the metabolites listed in Table S5; Figure S10: Reconstructed ion chromatograms of *m*/*z* 304.1856, *m*/*z* 320.1856, and *m*/*z* 336.1805 after analysis of one QC sample of rat urine in full scan in positive ionization mode using hydrophilic interaction chromatography (HILIC). Metabolite identification numbers (M) match with the metabolites listed in Table S5.
