# Supplementary material for: In Vitro and In Vivo Toxicometabolomics of the Synthetic Cathinone PCYP Studied by Means of LC-HRMS/MS
Source: Metabolites. 2022 Dec 2;12(12):1209. doi: 10.3390/metabo12121209 (PMC9783153; doi:10.3390/metabo12121209)
Supplement: Supplementary file 1 [file metabolites-12-01209-s001.zip › metabolites-2071856-supplementary.pdf]

**Supporting Information**

# In Vitro and In Vivo Toxicometabolomics of the Synthetic Cathinone PCYP Studied by Means of LC-HRMS/MS

Selina Hemmer[1], Lea Wagmann[1], Benedikt Pulver[2], Folker Westphal[2], Markus R. Meyer[1,*]

[1]Department of Experimental and Clinical Toxicology, Institute of Experimental and Clinical Pharmacology and Toxicology, Center for Molecular Signaling (PZMS), Saarland University, Homburg, Germany

[2]State Bureau of Criminal Investigation Schleswig-Holstein, Kiel, Germany

**Corresponding author**

*Markus R. Meyer, email: markus.meyer@uks.eu

**Table S1.** Overview of the peak picking and alignment parameters used for preprocessing for the reversed-phase (RP) and hydrophilic interaction chromatography (HILIC) column and the respective matrices. Pos = positive, neg = negative, ppm = allowed ppm deviation of mass traces for peak picking, snthresh = signal to noise threshold, mzdiff = minimum difference in *m/z* for two peaks to be considered as separate, prefilter 1 = minimum of scan points, prefilter 2 = minimum abundance, bw = bandwidth for grouping of peaks across separate chromatograms.

| Column | Matrix | Polarity | Peak width, min | Peak width, max | ppm | sntresh | mzdiff | Prefilter 1 | Prefilter 2 | bw |
|---|---|---|---|---|---|---|---|---|---|---|
| RP | pHLM | pos | 8.9 | 100 | 1.8 | 10 | 0.018 | 7 | 100 | 5.0 |
| | | neg | 8.9 | 15 | 1.7 | 27 | 0.094 | 5 | 100 | 1.0 |
| | Urine | pos | 8.9 | 19 | 1.0 | 12 | 0.012 | 7 | 100 | 2.5 |
| | | neg | 7.8 | 15 | 2.5 | 18 | -0.098 | 6 | 100 | 4.5 |
| | Plasma | pos | 8.9 | 33 | 1.3 | 12 | 0.1 | 7 | 100 | 1.0 |
| | | neg | 6.8 | 100 | 1.8 | 16 | 0.01 | 5 | 100 | 1.0 |
| HILIC | pHLM | pos | 7.8 | 29 | 1.6 | 17 | 0.006 | 6 | 100 | 0.5 |
| | | neg | 7.8 | 17 | 2.5 | 51 | 0.01 | 6 | 1300 | 1.0 |
| | Urine | pos | 8.9 | 21 | 1.9 | 16 | 0.02 | 8 | 100 | 1.5 |
| | | neg | 8.9 | 35 | 1.3 | 15 | 0.022 | 8 | 100 | 1.5 |
| | Plasma | pos | 8.9 | 46 | 1.4 | 6 | 0.034 | 6 | 100 | 0.2 |
| | | neg | 8.9 | 25 | 2.5 | 15 | 0.034 | 6 | 100 | 0.9 |

**Table S2.** Overview of the significant features using reversed-phase (RP) and hydrophilic interaction chromatography (HILIC) column in pooled human liver microsome incubation. Features are sorted according to *m/z* values, followed by the polarity, the retention time (RT) for the corresponding column in seconds (sec), identity, and the identification level according to MSI. Hyphen (-) means that the feature was not significant using the corresponding column.

| *m/z* | Polarity | RP RT, sec | HILIC RT, sec | Identity | Identification level according to MSI |
|---|---|---|---|---|---|
| 105.0331 | Positive | - | 192 | PCYP artifact | 3 |
| 146.0812 | Positive | - | 61 | Unknown | 4 |
| 158.0812 | Positive | - | 83, 126 | Unknown | 4 |
| 176.0918 | Positive | 31 | 126 | Unknown | 4 |
| 218.1539 | Positive | - | 224 | PCYP-M (*N*-dealkyl-) | 3 |
| 220.1696 | Positive | - | 247 | Unknown | 4 |
| 271.1884 | Positive | 314 | - | PCYP-M (dehydro-) isotope | 3 |
| 272.2008 | Positive | 314 | 192 | PCYP | 1 |
| 273.2041 | Positive | 314 | 192 | PCYP isotope | 3 |
| 274.2164 | Positive | 314 | 195 | PCYP isotope | 3 |
| 275.2197 | Positive | 314 | 194 | PCYP isotope | 3 |
| 276.223 | Positive | 314 | - | PCYP isotope | 3 |
| 286.1801 | Positive | 214, 235 | - | PCYP-M (oxo-) | 3 |
| 288.1957 | Positive | 280, 302, 255 | 222, 259, 244 | PCYP-M (hydroxy-) | 3 |
| 289.199 | Positive | - | 189, 222, 259, 244 | PCYP-M (hydroxy-) isotope | 3 |
| 290.2113 | Positive | 306 | 266 | PCYP-M (ring opened hydroxy-) | 3 |
| 291.2146 | Positive | - | 205 | PCYP-M (ring opened hydroxy-) isotope | 3 |
| 304.1907 | Positive | 202, 309 | 234 | PCYP-M (dihydroxy-) | 3 |
| 305.1939 | Positive | 309 | 234, 252 | PCYP-M (dihydroxy-) isotope | 3 |
| 306.2063 | Positive | 216 | - | PCYP-M (ring opened dihydroxy) | 3 |
| 566.5508 | Positive | - | 42 | Unknown | 4 |

**Table S3.** Overview of the significant features using reversed-phase (RP) and hydrophilic interaction chromatography (HILIC) column in rat plasma. Features are sorted according to *m/z* values, followed by the polarity, the retention time (RT) for the corresponding column in seconds (sec), identity, and the identification level according to MSI. Hyphen (-) means that the feature was not significant using the corresponding column.

| *m/z* | Polarity | RP RT, sec | HILIC RT, sec | Identity | Identification level according to MSI |
|---|---|---|---|---|---|
| 146.0599 | Positive | - | 71 | Quinolin-2-ol | 2 (NIST msms) |
| 189.0579 | Positive | - | 66 | 3-Methyladipic acid $[M+H+H_2O]^+$ | 2 (NIST msms) |
| 190.0613 | Positive | - | 67 | 3-Methyladipic acid $[M+H+H_2O]^+$ isotope | 2 (NIST msms) |
| 268.1038 | Positive | 35,61 | 248 | Adenosine | 2 (NIST msms) |
| 269.0878 | Positive | - | 330 | Unknown | 4 |
| 276.2685 | Positive | - | 178 | Unknown | 4 |
| 291.2721 | Positive | 390 | - | Unknown | 4 |
| 304.1904 | Positive | 306 | 235 | PCYP-M (dihydroxy -) | 3 |
| 305.1939 | Positive | 306 | 235 | PCYP-M (dihydroxy-) isotope | 3 |
| 309.1010 | Negative | - | 215 | Unknown | 4 |
| 310.1493 | Positive | 167 | - | Unknown | 4 |
| 312.0945 | Negative | - | 248 | Unknown | 4 |
| 318.1699 | Positive | - | 237 | PCYP-M (dihydroxy-, oxo) | 3 |
| 320.1856 | Positive | 215 | 290 | PCYP-M (trihydroxy-) | 3 |
| 321.0432 | Negative | - | 206 | Unknown | 4 |
| 321.1886 | Positive | 215 | - | PCYP-M (trihydroxy-) isotope | 3 |
| 328.3845 | Positive | - | 160 | Unknown | 4 |
| 416.3740 | Negative | - | 159 | Unknown | 4 |
| 562.5880 | Negative | 24 | - | Unknown | 4 |

**Table S4.** Overview of the significant features using reversed-phase (RP) and hydrophilic interaction chromatography (HILIC) column in rat urine. Features are sorted according to *m/z* values, followed by the polarity, the retention time (RT) for the corresponding column in seconds (sec), identity, and the identification level according to MSI. Hyphen (-) means that the feature was not significant using the corresponding column.

| *m/z* | Polarity | RP RT, sec | HILIC RT, sec | Identity | Identification level according to MSI |
|---|---|---|---|---|---|
| 146.0602 | Positive | 244 | 70 | Quinolin-2-ol | 2 (NIST msms) |
| 147.0635 | Positive | 244 | - | Quinolin-2-ol isotope | 2 (NIST msms) |
| 148.0965 | Positive | - | 456 | Unknown | 4 |
| 162.0551 | Positive | - | 230 | Dihydroxyquinoline | 3 (NIST msms) |
| 163.0584 | Positive | - | 230 | Dihydroxyquinoline isotope | 3 (NIST msms) |
| 185.0437 | Positive | - | 303 | Kynurenic acid $[M\text{-}CH_2O_2\text{+}Na]^+$ | 2 (massbank) |
| 186.0470 | Positive | - | 303 | Kynurenic acid $[M\text{-}CH_2O_2\text{+}Na]^+$ isotope | 2 (massbank) |
| 189.0582 | Positive | 156 | 67 | Unknown | 4 |
| 190.0503 | Negative | - | 176 | Unknown | 4 |
| 190.0614 | Positive | - | 67 | Unknown isotope | 4 |
| 208.1183 | Negative | - | 398 | Unknown | 4 |
| 208.4951 | Negative | - | 320 | Unknown | 4 |
| 208.9913 | Negative | - | 320 | Unknown | 4 |
| 211.0401 | Positive | 156 | - | Unknown | 4 |
| 215.0013 | Negative | - | 301 | Unknown | 4 |
| 219.9996 | Negative | - | 248 | Unknown | 4 |
| 220.9920 | Negative | - | 248 | Unknown | 4 |
| 221.0448 | Negative | 226 | - | Unknown | 4 |
| 239.9966 | Negative | 203, 151 | 232, 271 | Unknown | 4 |
| 240.0539 | Positive | - | 263 | Unknown | 4 |
| 242.0118 | Positive | 152 | 271 | Unknown | 4 |
| 242.0122 | Negative | - | 245 | Unknown | 4 |
| 242.0123 | Negative | 157 | - | Unknown | 4 |

**Table S4.** Continued.

| *m/z* | Polarity | RP RT, sec | HILIC RT, sec | Identity | Identification level according to MSI |
|---|---|---|---|---|---|
| 243.0977 | Positive | - | 472 | Unknown | 4 |
| 243.1817 | Positive | - | 425 | Unknown | 4 |
| 245.0924 | Negative | - | 242 | Unknown | 4 |
| 247.9776 | Negative | 238 | - | Unknown | 4 |
| 250.1439 | Positive | - | 339 | PCYP artifact | 3 |
| 255.0653 | Positive | - | 225 | Daidzein | 2 (massbank) |
| 260.0588 | Positive | 186 | - | Unknown | 4 |
| 270.0483 | Negative | 336 | - | Unknown | 4 |
| 271.0390 | Negative | - | 226 | Unknown | 4 |
| 271.0819 | Negative | - | 107 | Unknown | 4 |
| 281.1136 | Negative | 266 | 329 | Unknown | 4 |
| 283.1290 | Positive | 266 | - | Unknown | 4 |
| 283.9306 | Negative | 207, 238 | - | Unknown | 4 |
| 284.1242 | Positive | - | 468 | Unknown | 4 |
| 285.2286 | Positive | - | 456 | Unknown | 4 |
| 285.8645 | Negative | 266 | - | Unknown | 4 |
| 286.0793 | Positive | - | 455 | Unknown | 4 |
| 287.1139 | Positive | 208 | - | Unknown | 4 |
| 288.0901 | Positive | 218 | 247 | Unknown | 4 |
| 288.1957 | Positive | - | 222 | PCYP-M (hydroxy-) | 3 |
| 289.0324 | Negative | - | 354 | Unknown | 4 |
| 290.9998 | Negative | - | 246 | Unknown | 4 |
| 297.0973 | Negative | - | 148 | Unknown | 4 |
| 299.8808 | Negative | 122 | - | Unknown | 4 |
| 302.1422 | Positive | - | 68 | Unknown | 4 |

**Table S4.** Continued.

| *m/z* | Polarity | RP RT, sec | HILIC RT, sec | Identity | Identification level according to MSI |
|---|---|---|---|---|---|
| 302.2108 | Positive | - | 366 | Unknown | 4 |
| 303.1704 | Positive | - | 271 | Unknown | 4 |
| 303.2109 | Positive | - | 366 | Unknown isotope | 4 |
| 304.0070 | Negative | - | 320 | Unknown | 4 |
| 304.1910 | Positive | 305 | 232 | PCYP-M (dihydroxy-) | 3 |
| 305.1942 | Positive | 305 | - | PCYP-M (dihydroxy-) isotope | 3 |
| 306.1701 | Positive | 204 | 296 | PCYP-M (hydroxy + pyrrolidin cleavage with oxidation to COOH) | 3 |
| 307.0578 | Positive | 312 | - | Unknown | 4 |
| 307.0749 | Positive | 249 | - | Unknown | 4 |
| 309.0067 | Negative | - | 247 | Unknown | 4 |
| 310.0720 | Positive | 218 | 247 | Unknown | 4 |
| 311.2119 | Positive | 320 | 218 | Unknown | 4 |
| 312.2151 | Positive | - | 218 | Unknown isotope | 4 |
| 316.1546 | Negative | - | 236 | Unknown | 4 |
| 317.0329 | Negative | - | 241 | Unknown | 4 |
| 318.1702 | Positive | 222 | 195, 233, 318 | PCYP-M (dihydroxy-, oxo) | 3 |
| 319.1266 | Positive | 245 | - | Unknown | 4 |
| 319.1734 | Positive | 222 | 233, 318 | PCYP-M (dihydroxy-, oxo) isotope | 3 |
| 320.1859 | Positive | 214, 232 | 264, 302 | PCYP-M (trihydroxy-) | 3 |
| 321.1892 | Positive | 214, 232 | 302 | PCYP-M (trihydroxy-) isotope | 3 |
| 322.2015 | Positive | 193 | - | Unknown | 4 |
| 323.2048 | Positive | 192 | 296 | Unknown isotope | 4 |

**Table S4.** Continued

| *m/z* | Polarity | RP<br>RT, sec | HILIC<br>RT, sec | Identity | Identification level according to MSI |
|---|---|---|---|---|---|
| 324.9200 | Negative | - | 234 | Unknown | 4 |
| 325.0855 | Positive | 206 | 305 | Unknown | 4 |
| 326.0460 | Positive | - | 246 | Unknown | 4 |
| 327.1079 | Negative | - | 176 | Unknown | 4 |
| 327.2069 | Positive | 243 | 273 | Unknown | 4 |
| 331.0851 | Negative | - | 235 | Unknown | 4 |
| 332.1491 | Positive | - | 86 | Unknown | 4 |
| 334.0101 | Negative | - | 226 | Unknown | 4 |
| 334.1108 | Positive | - | 69 | Unknown | 4 |
| 334.1651 | Positive | 188, 248 | 115, 324 | PCYP-M (trihydroxy-, oxo) | 3 |
| 335.0223 | Positive | - | 324 | PCYP-M (trihydroxy-, oxo) isotope | 3 |
| 335.9012 | Negative | 235 | - | Unknown | 4 |
| 336.1807 | Positive | 176, 197 | 323, 341 | PCYP-M (tetrahydroxy-) | 3 |
| 337.1807 | Positive | 176 | 323, 341 | PCYP-M (tetrahydroxy-) isotope | 3 |
| 338.0414 | Negative | - | 241 | Unknown | 4 |
| 341.1861 | Positive | 210 | 375 | Unknown | 4 |
| 343.2019 | Positive | - | 321 | Unknown | 4 |
| 343.8885 | Negative | 182 | - | Unknown | 4 |
| 346.1433 | Positive | - | 245 | Unknown | 4 |
| 347.1466 | Positive | - | 245 | Unknown | 4 |
| 347.2541 | Positive | - | 374 | Unknown | 4 |
| 349.0703 | Negative | - | 248 | Unknown | 4 |
| 351.0858 | Positive | - | 114 | Unknown | 4 |
| 352.0487 | Positive | - | 225 | Unknown | 4 |

**Table S4.** Continued.

| *m/z* | Polarity | RP RT, sec | HILIC RT, sec | Identity | Identification level according to MSI |
|---|---|---|---|---|---|
| 353.0329 | Negative | - | 245 | Unknown | 4 |
| 356.1471 | Positive | 247 | - | Unknown | 4 |
| 360.1920 | Positive | - | 202, 344 | Unknown | 4 |
| 361.1952 | Positive | - | 344 | Unknown isotope | 4 |
| 365.2357 | Negative | - | 146 | Unknown | 4 |
| 367.0484 | Negative | - | 240 | Unknown | 4 |
| 371.1338 | Negative | 225 | - | Unknown | 4 |
| 372.1212 | Positive | - | 114 | Unknown | 4 |
| 376.1426 | Positive | 271 | - | Unknown | 4 |
| 380.0548 | Negative | - | 246 | Unknown | 4 |
| 390.1762 | Positive | 225 | - | Unknown | 4 |
| 391.0682 | Negative | - | 223 | Unknown | 4 |
| 426.1333 | Positive | - | 247 | Unknown | 4 |
| 462.0523 | Negative | - | 320 | Unknown | 4 |
| 464.2285 | Positive | - | 393 | PCYP-M (hydroxy-glucuronide-) | 3 |
| 465.2155 | Positive | 317 | 360 | Unknown | 4 |
| 466.2189 | Positive | - | 360 | Unknown isotope | 4 |
| 573.3299 | Negative | - | 139 | Unknown | 4 |

**Table S5.** Detected PCYP metabolites using reversed-phase (RP) and hydrophilic interaction chromatography (HILIC) column in their corresponding matrices namely pooled human liver microsomes (H), rat urine (U), and rat plasma (P) in which the metabolites could be detected. Metabolite identification numbers (ID) match with the labeling of the structure in Figure 1. For each metabolite the calculated exact mass of the protonated molecule and elemental composition are given. Hyphen (-) means that the metabolite was not significant in any matrix of the respective column.

| Metabolite-ID | Calculated exact mass, *m/z* | Elemental composition | RP | HILIC |
|---|---|---|---|---|
| PCYP | 272.2009 | $C_{18}H_{25}NO$ | H | H |
| M1 | 288.1958 | $C_{18}H_{25}NO_2$ | H | H |
| M2 | 288.1958 | $C_{18}H_{25}NO_2$ | H | H |
| M3 | 288.1958 | $C_{18}H_{25}NO_2$ | H | H, U |
| M4 | 218.1539 | $C_{14}H_{19}NO$ | - | H |
| M5 | 304.1907 | $C_{18}H_{25}NO_3$ | H, U, P | H, U, P |
| M6 | 320.1856 | $C_{18}H_{25}NO_4$ | U, P | U, P |
| M7 | 336.1805 | $C_{18}H_{25}NO_5$ | U | U |
| M8 | 286.1802 | $C_{18}H_{23}NO_2$ | H | - |
| M9 | 318.1700 | $C_{18}H_{23}NO_3$ | U, P | U, P |
| M10 | 334.1649 | $C_{18}H_{25}NO_5$ | U | U |
| M11 | 290.2115 | $C_{18}H_{27}NO_2$ | H | H |
| M12 | 306.2064 | $C_{18}H_{27}NO_3$ | H | - |
| M13 | 304.1907 | $C_{18}H_{25}NO_3$ | H | - |
| M14 | 320.1856 | $C_{18}H_{25}NO_4$ | U | U |
| M15 | 306.1700 | $C_{17}H_{23}NO_4$ | U | U |
| M16 | 250.1438 | $C_{14}H_{19}NO_3$ | - | U |
| M17 | 464.2279 | $C_{24}H_{33}NO_8$ | - | U |

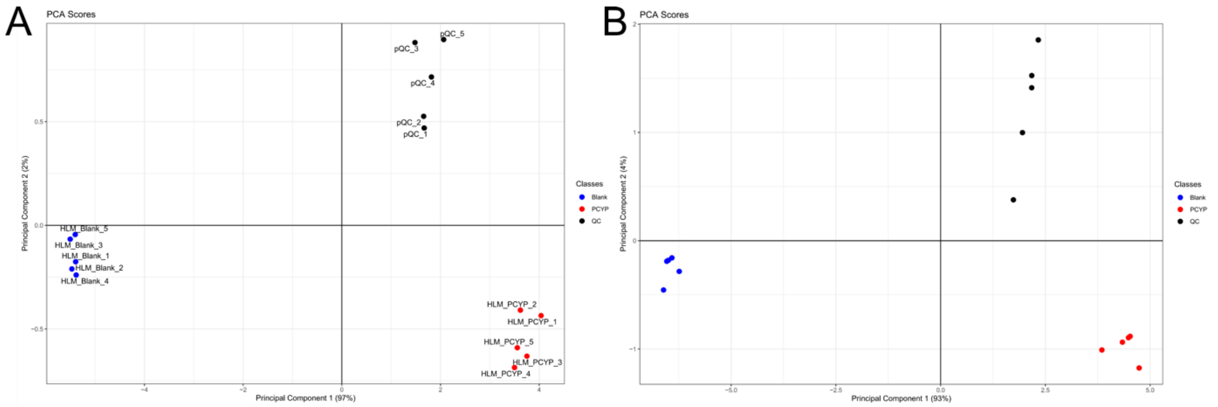


**Figure S1.** Results of scores of principal component analysis of pooled human liver microsome samples after analysis using reversed-phase (RP) and hydrophilic interaction chromatography (HILIC) in positive ionization mode. A = RP pos, B = HILIC pos.

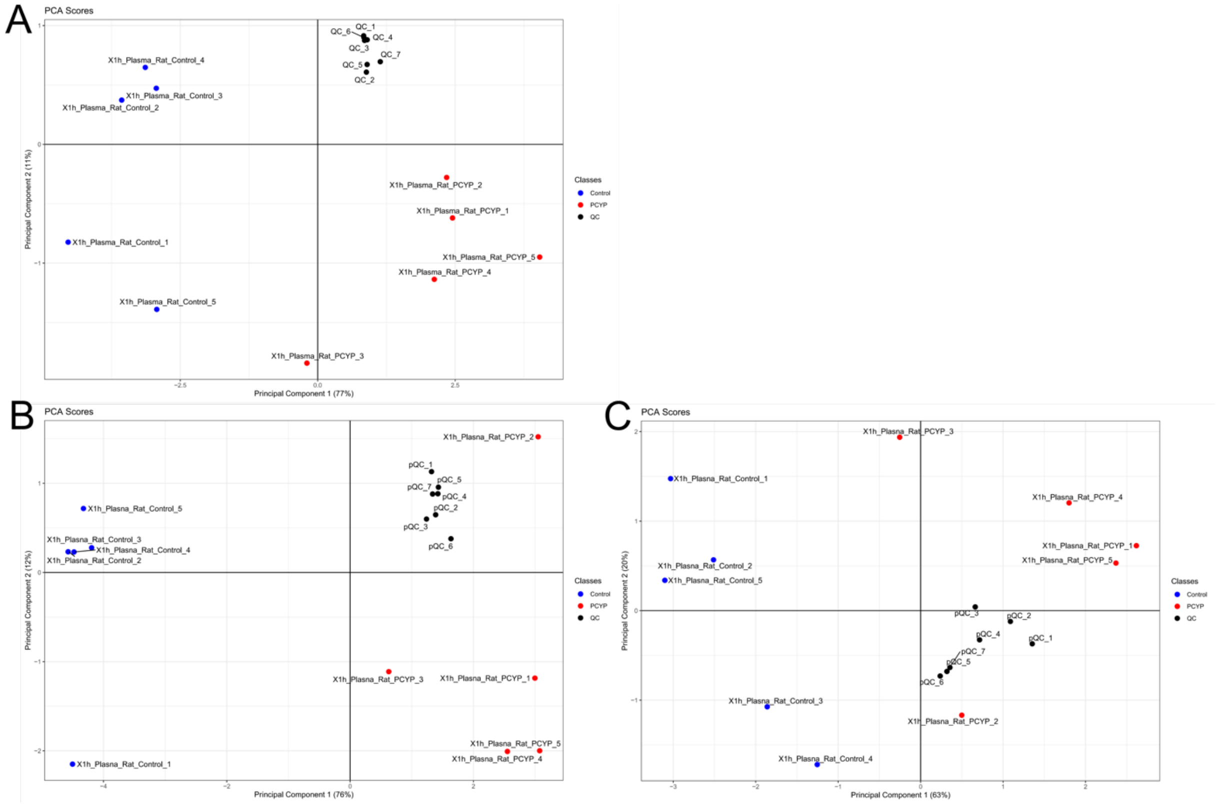


**Figure S2.** Results of scores of principal component analysis of rat plasma samples after analysis using reversed-phase (RP) and hydrophilic interaction chromatography (HILIC) in positive and negative ionization mode. A = PH pos, B = HILIC pos, C = HILIC neg.

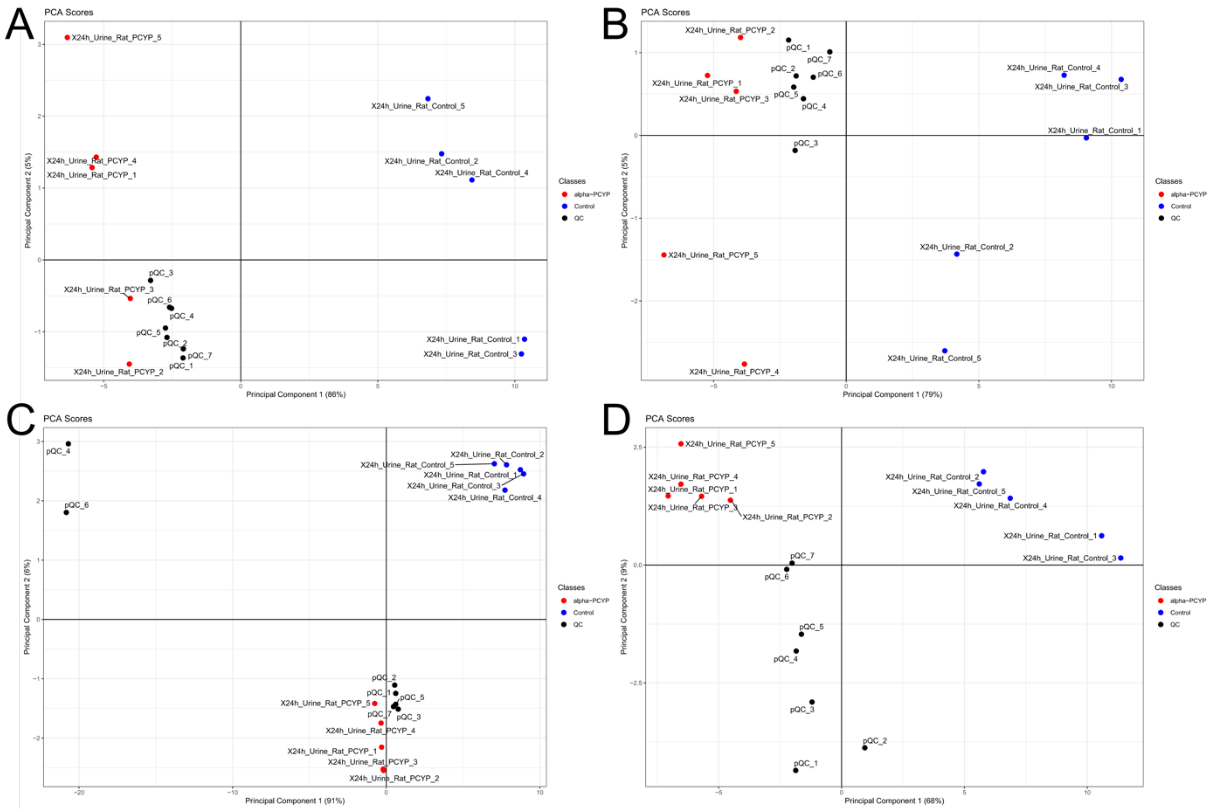


**Figure S3.** Results of scores of principal component analysis of rat urine samples after analysis using reversed-phase (RP) and hydrophilic interaction chromatography (HILIC) in positive and negative ionization mode. A = RP pos, B = RP neg, C = HILIC pos, D = HILIC neg.

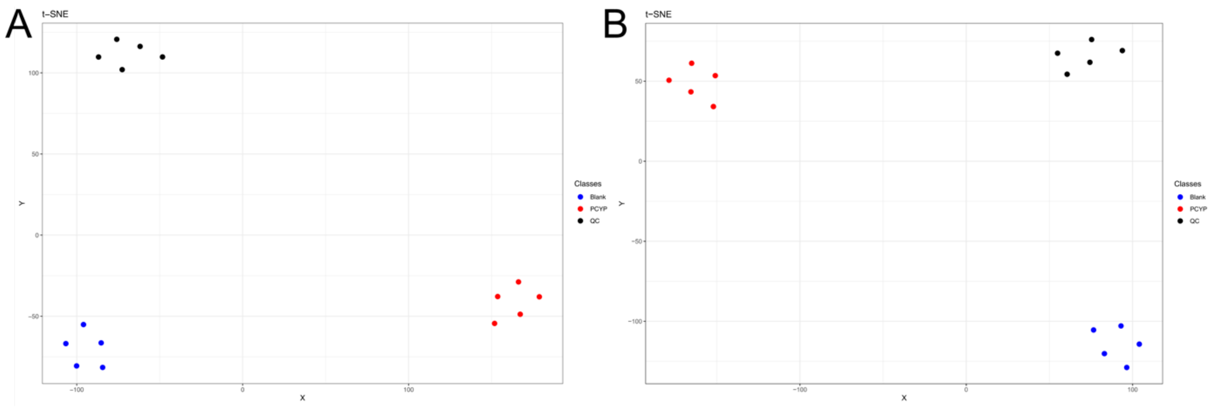


**Figure S4.** Results of t-distributed stochastic neighborhood embedding (t-SNE) of pooled human liver microsome samples after analysis using reversed-phase (RP) and hydrophilic interaction chromatography (HILIC) in positive ionization mode. A = RP pos, B = HILIC pos.

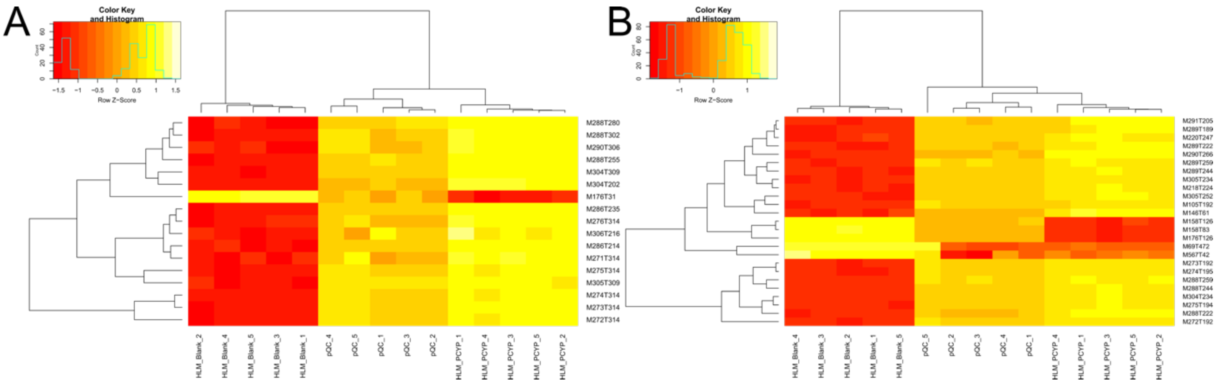


**Figure S5.** Results of heat map of hierarchical clustering of pooled human liver microsome samples after analysis using reversed-phase (RP) and hydrophilic interaction chromatography (HILIC) in positive ionization mode. A = RP pos, B = HILIC pos.

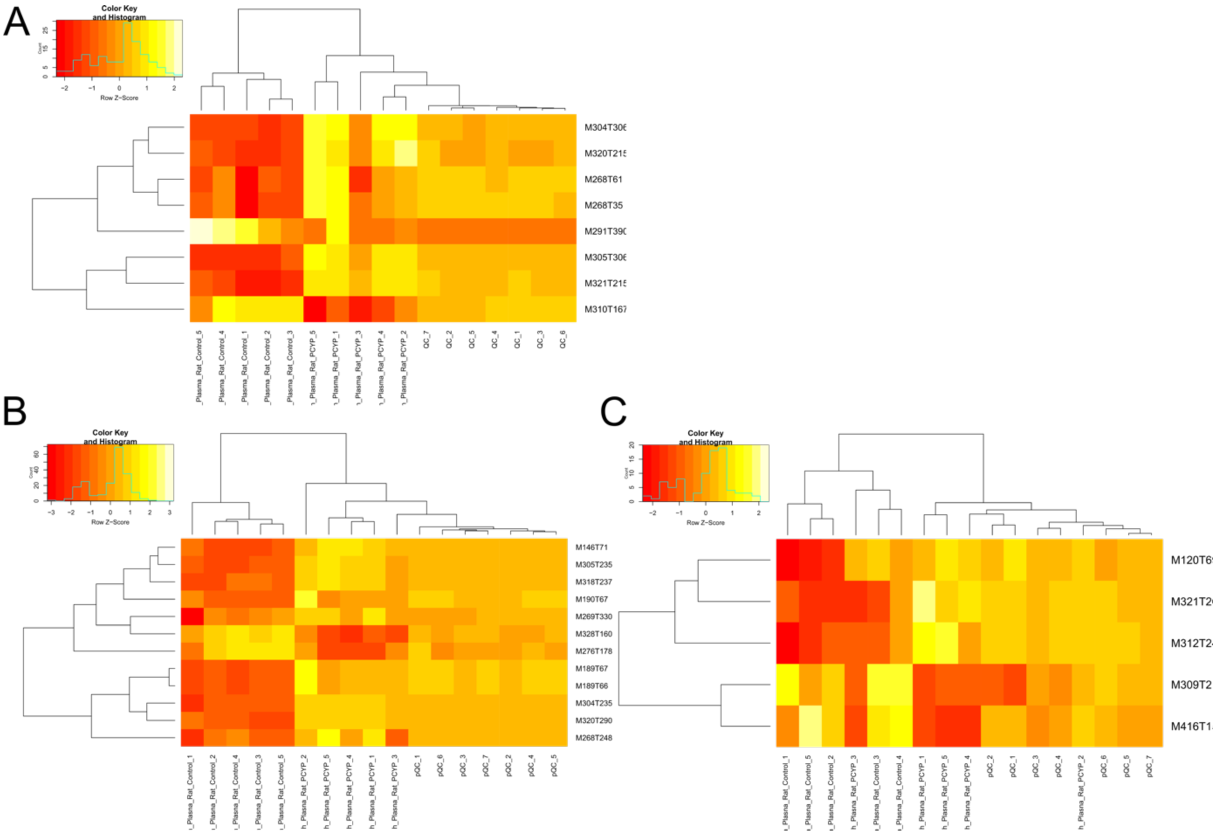


**Figure S6.** Results of heat map of hierarchical clustering of rat plasma samples after analysis using reversed-phase (RP) and hydrophilic interaction chromatography (HILIC) in positive and negative ionization mode. A = RP pos, B = HILIC pos, C = HILIC neg.

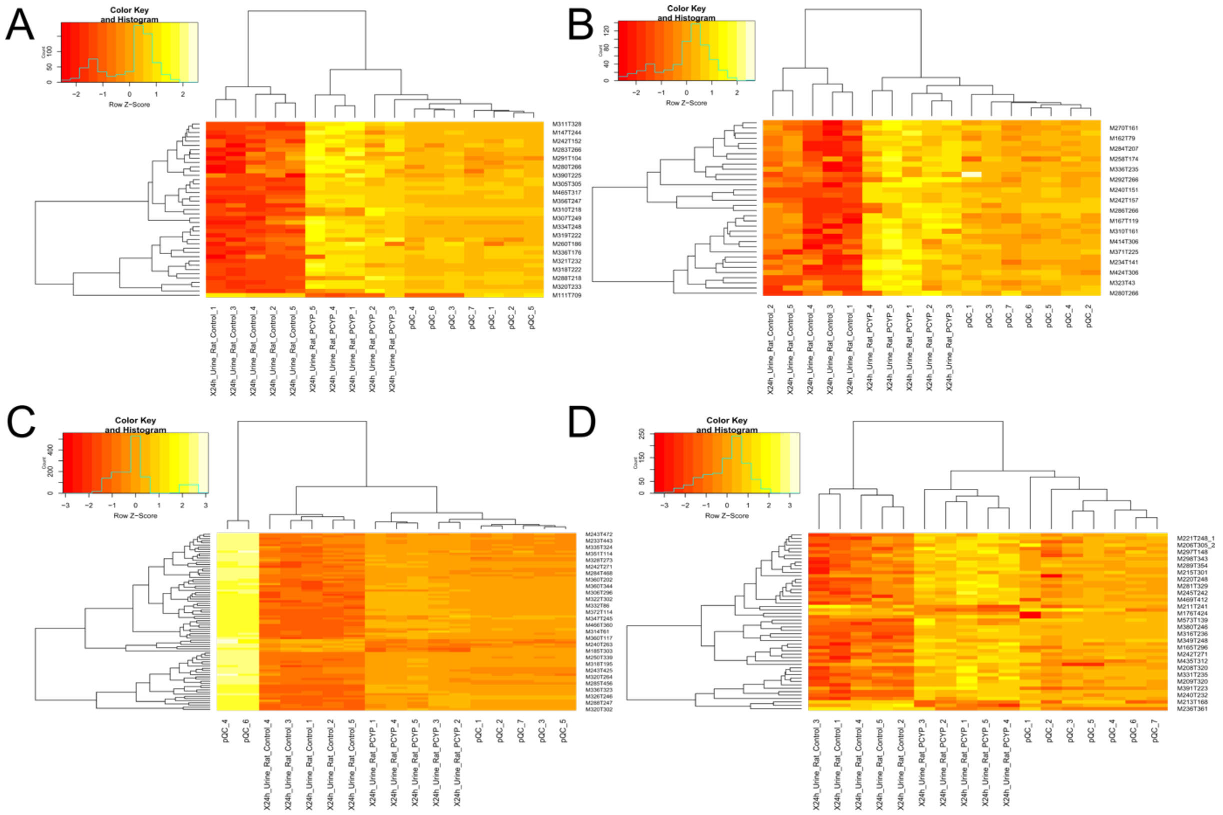


**Figure S7.** Results of heat map of hierarchical clustering of rat urine samples after analysis using reversed-phase (RP) and hydrophilic interaction chromatography (HILIC) in positive and negative ionization mode. A = RP pos, B = RP neg, C = HILIC pos, D = HILIC neg.

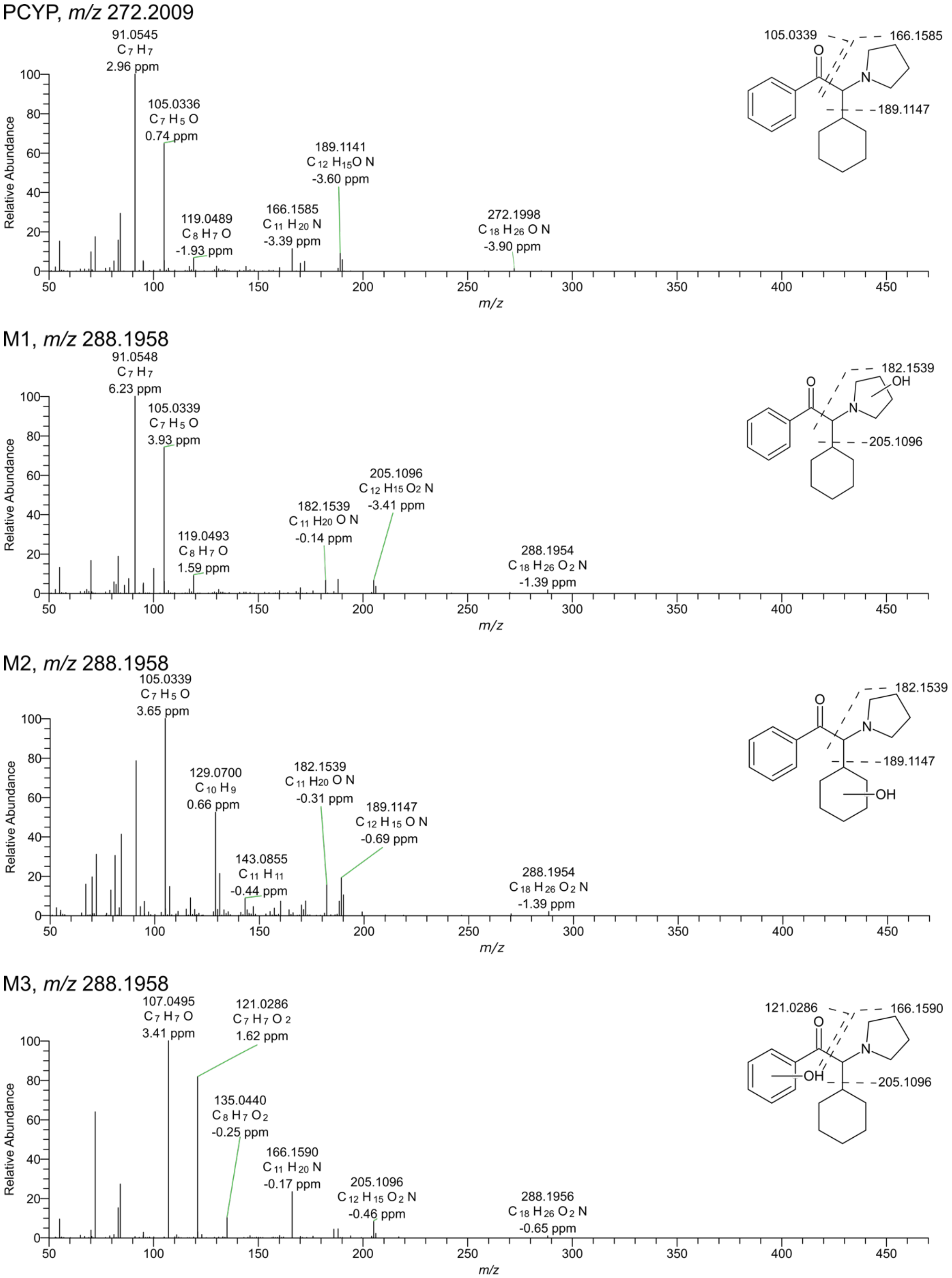


**Figure S8.** LC-HRMS/MS spectra of the PCYP metabolites detected in positive ionization mode. Fragments with accurate mass, calculated elemental formula, and mass error value in parts per million (ppm).

M4, *m/z* 218.1539

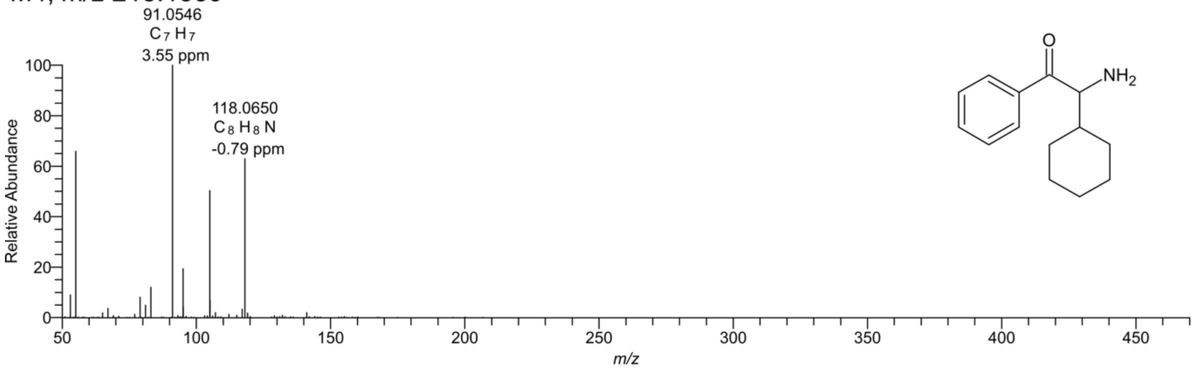


M5, *m/z* 304.1907

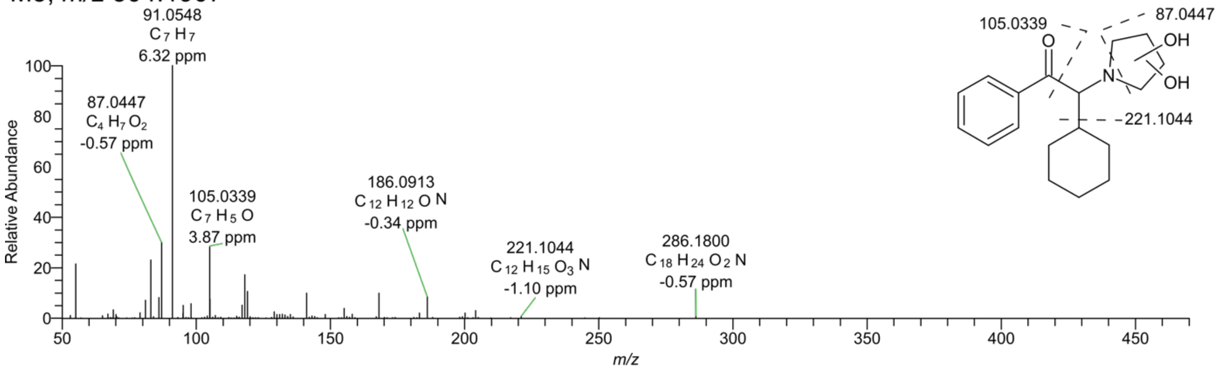


M6, *m/z* 320.1856

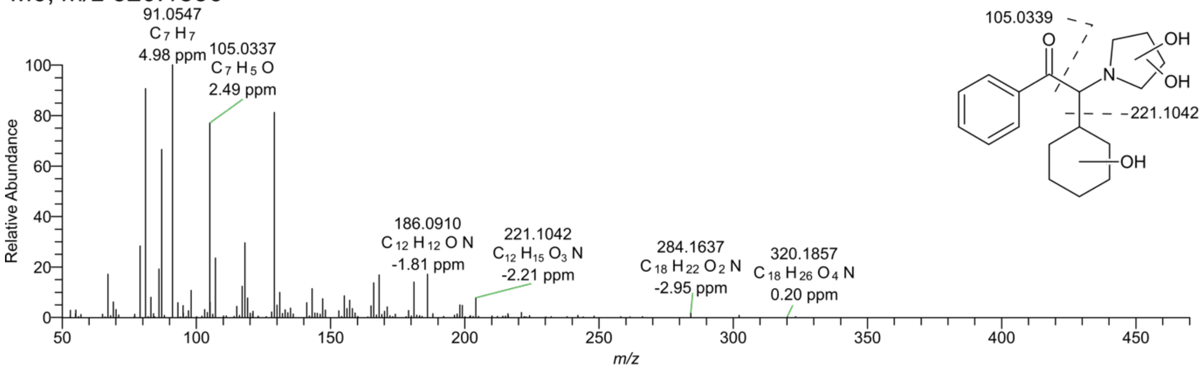


M7, *m/z* 336.1805

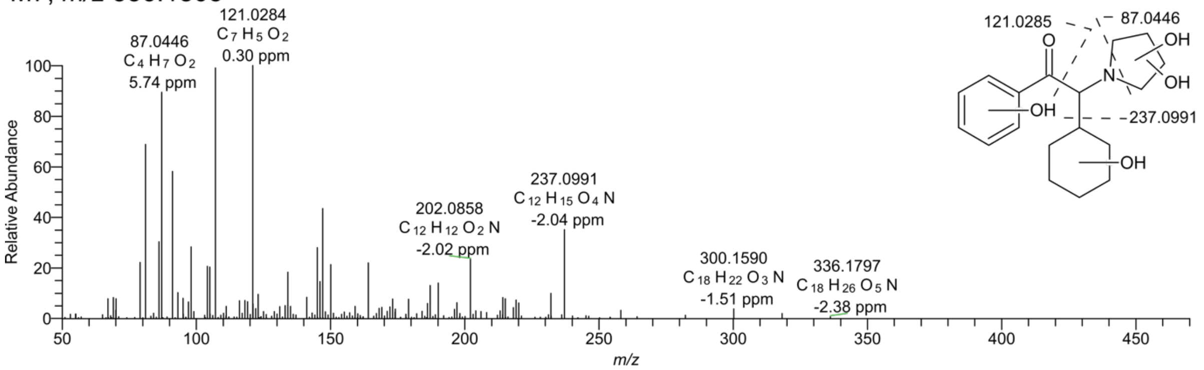


**Figure S8.** Continued.

M8, *m/z* 286.1802

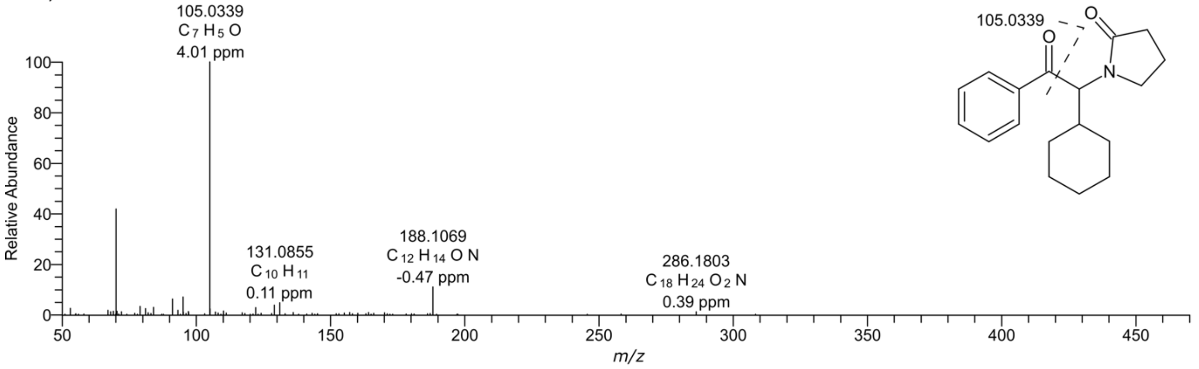


M9, *m/z* 318.1700

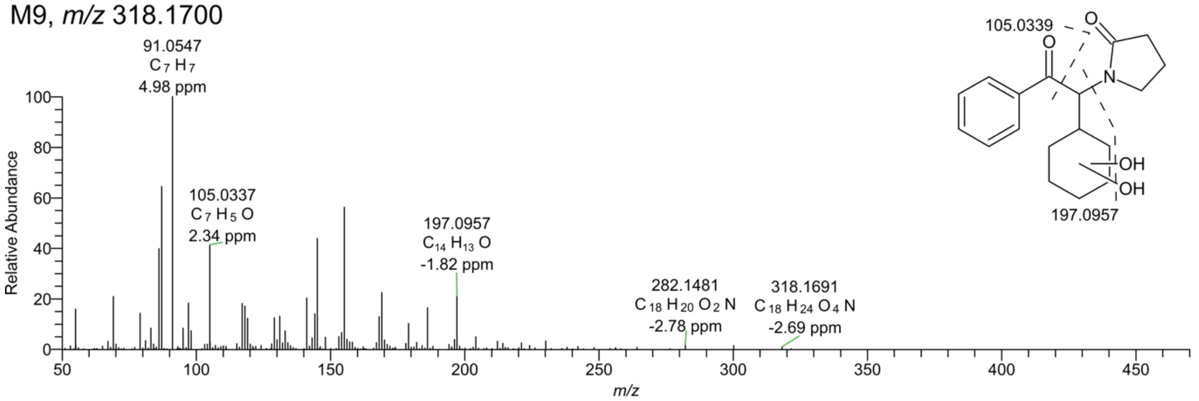


M10, *m/z* 334.1649

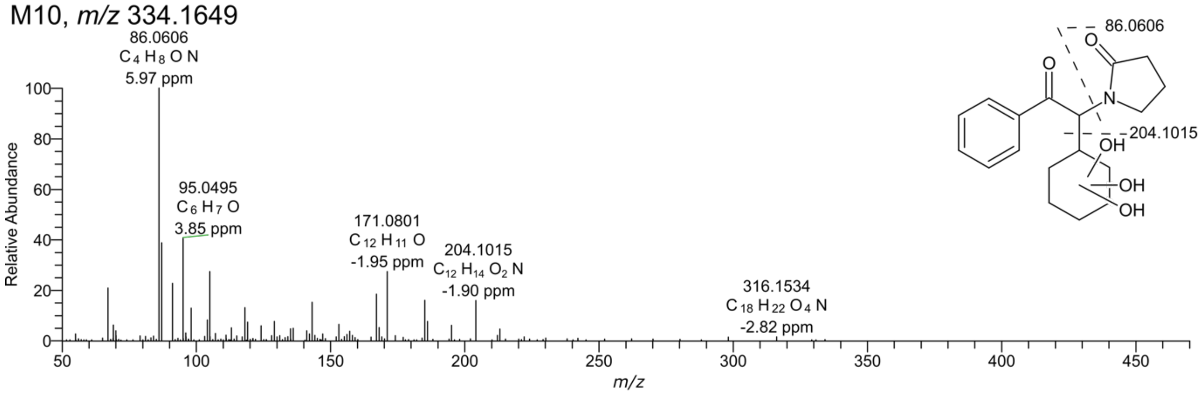


M11, *m/z* 290.2115

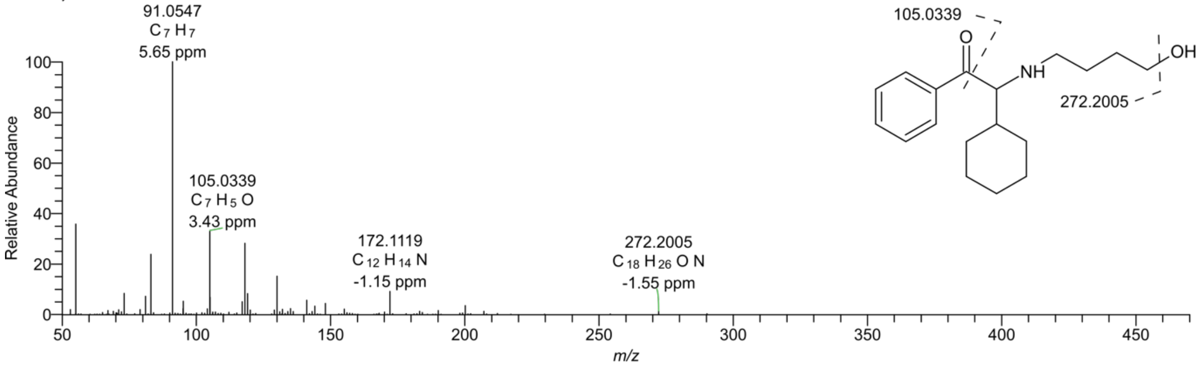


**Figure S8.** Continued.

M12, *m/z* 306.2064

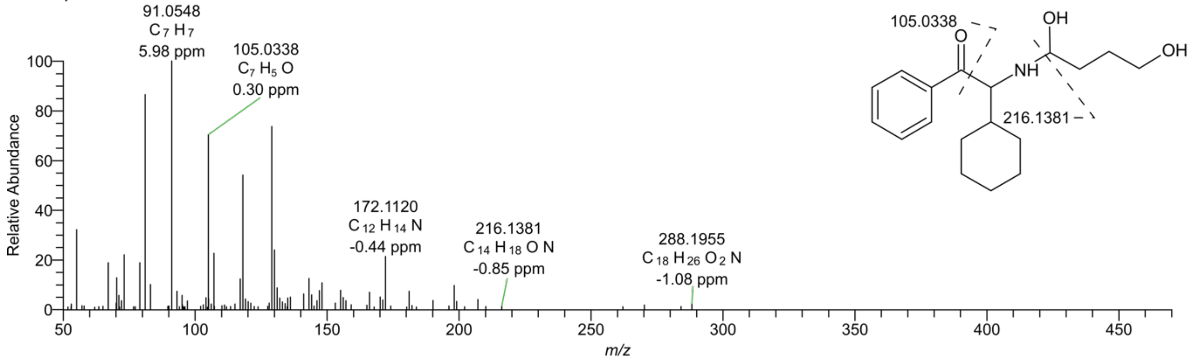


M13, *m/z* 304.1907

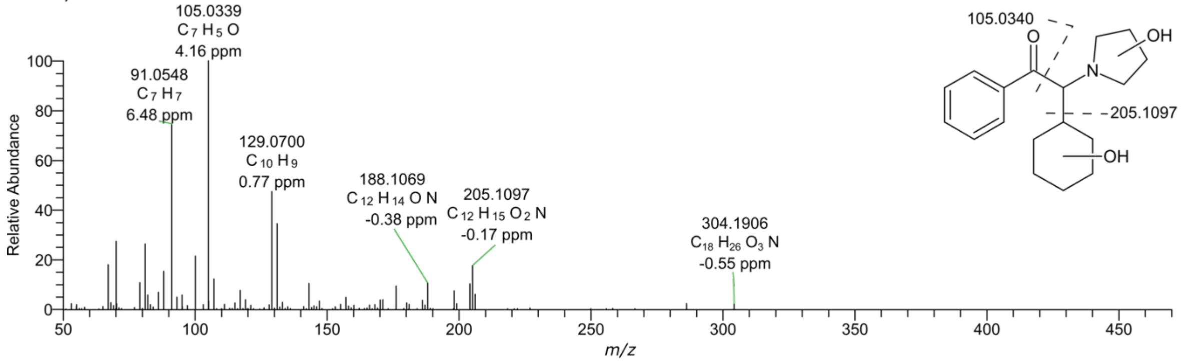


M14, *m/z* 320.1856

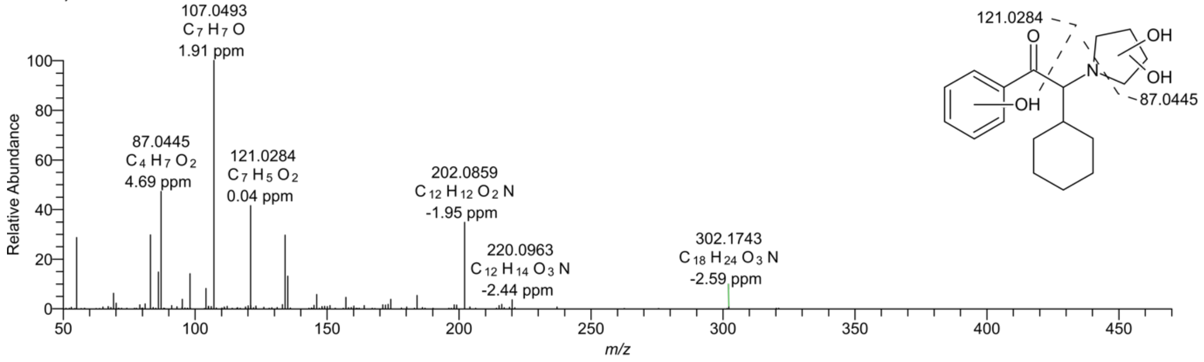


M15, *m/z* 306.1700

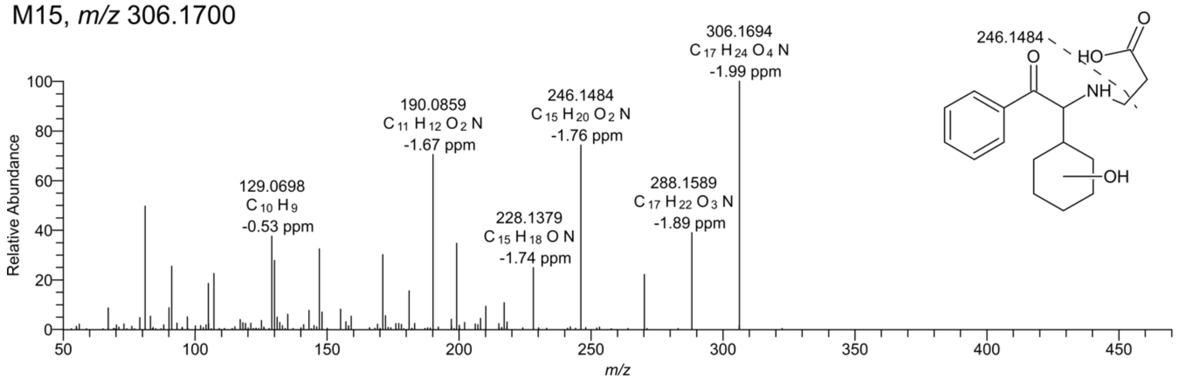


**Figure S8.** Continued.

M16, *m/z* 250.1438

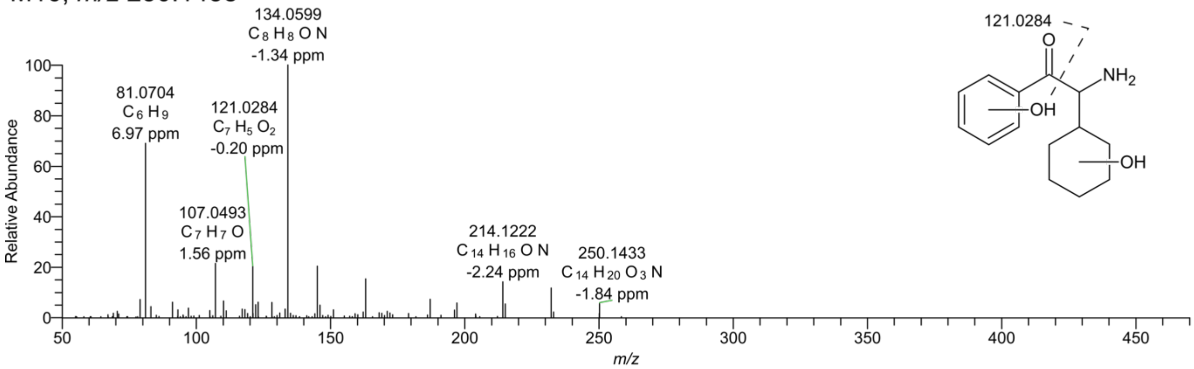


M17, *m/z* 464.2279

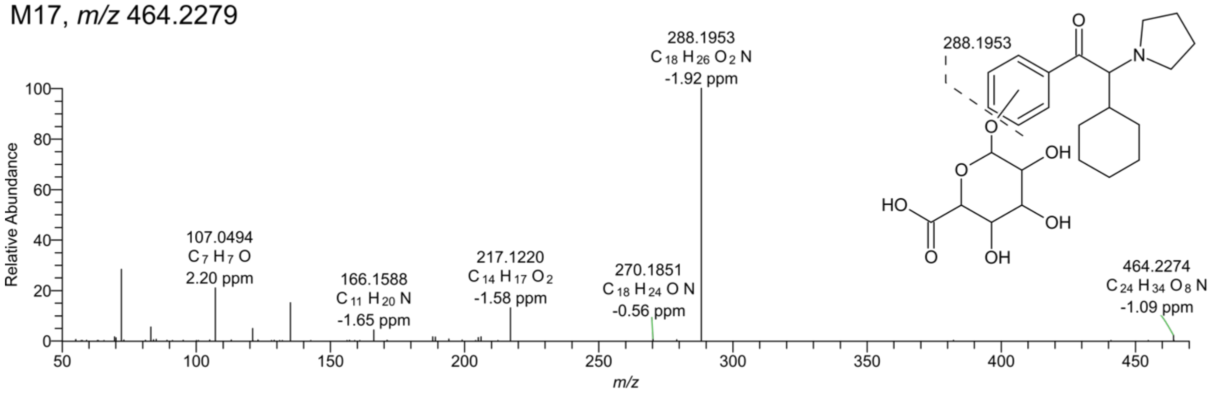


**Figure S8.** Continued.

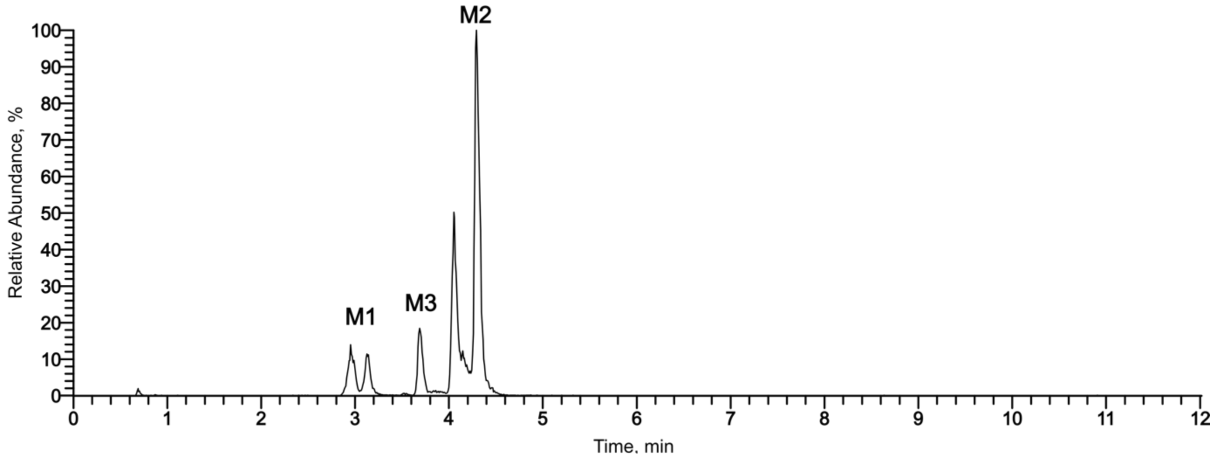


**Figure S9.** Reconstructed ion chromatogram of *m/z* 288.1958 after analysis of one QC sample of pooled human liver microsome in full scan in positive ionization mode using hydrophilic interaction chromatography (HILIC). Metabolite identification number (M) match with the metabolites listed in Table S5.

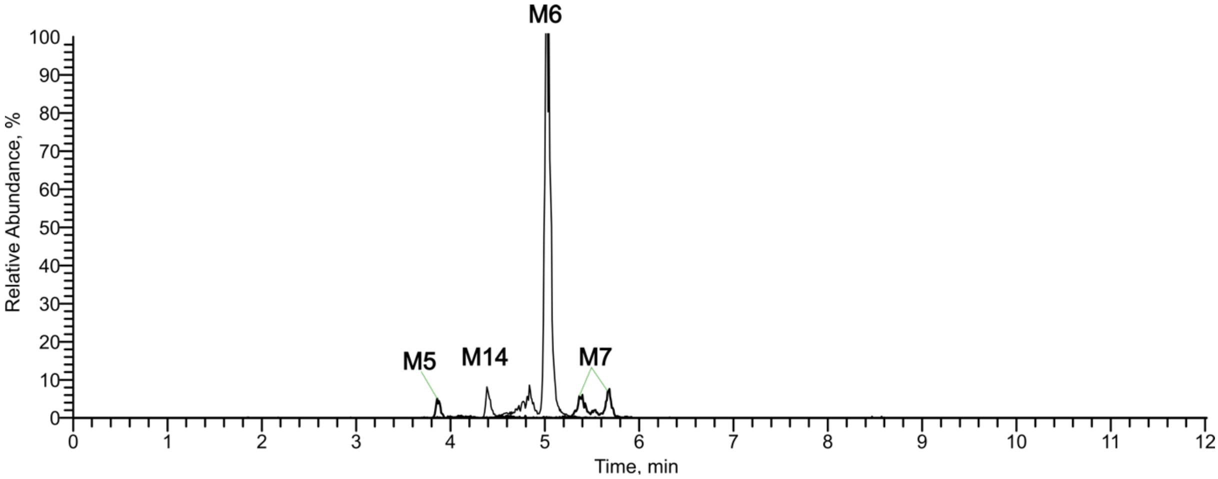


**Figure S10.** Reconstructed ion chromatograms of *m/z* 304.1856, *m/z* 320.1856, and *m/z* 336.1805 after analysis of one QC sample of rat urine in full scan in positive ionization mode using hydrophilic interaction chromatography (HILIC). Metabolite identification numbers (M) match with the metabolites listed in Table S5.
